# Ttyh1 Protein is Expressed in Glia In Vitro and Shows Elevated Expression in Activated Astrocytes Following *Status Epilepticus*

**DOI:** 10.1007/s11064-014-1455-3

**Published:** 2014-10-15

**Authors:** Elzbieta Wiernasz, Aleksandra Kaliszewska, Wojciech Brutkowski, Joanna Bednarczyk, Malgorzata Gorniak, Beata Kaza, Katarzyna Lukasiuk

**Affiliations:** 1 Laboratory of Epileptogenesis, The Nencki Institute of Experimental Biology, 3 Pasteur St, 02-093 Warsaw, Poland; 2 Laboratory of Imaging Tissue Structure and Function, The Nencki Institute of Experimental Biology, Warsaw, Poland; 3 Laboratory of Molecular Neurobiology, Neurobiology Center, The Nencki Institute of Experimental Biology, Warsaw, Poland

**Keywords:** Astrocytes, Gliosis, Microglia, Oligodendrocytes, *Status epilepticus*, Tweety

## Abstract

In a previous study, we showed that Ttyh1 protein is expressed in neurons in vitro and in vivo in the form of punctuate structures, which are localized to neuropil and neuronal somata. Herein, we provide the first description of Ttyh1 protein expression in astrocytes, oligodendrocytes and microglia in vitro. Moreover, using double immunofluorescence, we show Ttyh1 protein expression in activated astrocytes in the hippocampus following amygdala stimulation-induced *status epilepticus*. We demonstrate that in migrating astrocytes in in vitro wound model Ttyh1 concentrates at the edges of extending processes. These data suggest that Ttyh1 not only participates in shaping neuronal morphology, as previously described, but may also play a role in the function of activated glia in brain pathology. To localize Ttyh1 expression in the cellular compartments of neurons and astrocytes, we performed in vitro double immunofluorescent staining using markers for the following subcellular structures: endoplasmic reticulum (GRP78), Golgi apparatus (GM130), clathrin-coated vehicles (clathrin), early endosomes (Rab5 and APPL2), recycling endosomes (Rab11), trans-Golgi network (TGN46), endoplasmic reticulum membrane (calnexin), late endosomes and lysosomes (LAMP1) and synaptic vesicles (synaptoporin and synaptotagmin 1). We found that Ttyh1 is present in the endoplasmic reticulum, Golgi apparatus and clathrin-coated vesicles (clathrin) in both neurons and astrocytes and also in late endosomes or lysosomes in astrocytes. The presence of Ttyh1 was negligible in early endosomes, recycling endosomes, trans-Golgi network, endoplasmic reticulum membrane and synaptic vesicles.

## Introduction

Campbell et al. [1] first described the tweety (*twe*, *tty*) gene in the *fli* (flightless) genomic locus of *Drosophila* *melanogaster* [1]. Mutations of *tty* in *Drosophila* result in the loss of flight ability [1]. Homologs of the tweety (*tty*) gene have also been identified in *Caenorhabditis elegans* and mammals [2]. Three mammalian homologs, Ttyh1, Ttyh2 and Ttyh3, have been described [2–4].

Human and mouse homologs of the *D.* *melanogaster* tweety gene encode proteins predicted to be integral membrane proteins that share a high degree of sequence identity and common structural features with five or six transmembrane regions positioned in a similar arrangement. This arrangement comprises a pair of transmembrane regions, followed by a hydrophobic region and an additional pair of transmembrane regions, which are followed by more hydrophobic and transmembrane regions [2]. The glycosylated N-terminus is located extracellularly, whereas the involved in Ca^2+^ binding, an acid-residue-rich C-terminus is located cytoplasmically [5, 6]. The highest level of sequence variation among Tweety-related proteins is observed at the C-terminus, which likely specifies the function of these proteins [3].

The human Tweety proteins are putative maxi-chloride ion channels with differentially induced Cl^−^ channel activity [4, 7]. Tweety homolog 1 (Ttyh1) is a volume-regulated Cl^−^ channel, whose activity is regulated by cell swelling, whereas the activity of Ttyh2 and Ttyh3 is regulated by ionomycin and calcium ions [4, 7].

The expression of Ttyh1 mRNA is restricted to neural tissue and the testis [8]. In the brain, Ttyh1 mRNA has been detected in neurons [9, 10]. The expression of Ttyh1 protein is also neuronal and has been detected in neurons in vivo and in vitro [6, 8, 11]. The overexpression of Ttyh1 protein in cell lines and neurons in vitro leads to excessive filopodia formation [8, 11]. The involvement of Ttyh1 has been implicated in aberrant neuronal structural plasticity in vivo, as increased Ttyh1 protein expression was observed in the molecular layer of the dentate gyrus during epileptogenesis [11].

In a previous study, we have determined that Ttyh1 protein forms complexes with proteins localized in the endoplasmic reticulum and Golgi [11]. Thus, the aim of the present study is to verify these results using double immunocytochemistry with markers of different cellular compartments in vitro. In addition, we analyzed the expression of Ttyh1 in glial cells in vitro and in vivo in the normal and injured brain following *status epilepticus*. The results show the ubiquitous expression of Ttyh1 protein in astrocytes, oligodendrocytes and microglia in vitro and the increased expression of Ttyh1 in astrocytes in the hippocampus following *status epilepticus*. We also demonstrate that in migrating cultured astrocytes Ttyh1 is concentrated at the margins of extending processes.

## Materials and Methods

### Culture of Primary Hippocampal Neurons

Hippocampal neurons were cultured as previously described [11, 12]. Briefly, the hippocampi were dissected from E18 to E19 Wistar rat embryos and dissociated through incubation in 0.04 % trypsin (#27250-0180, Gibco). The cell suspension was plated onto poly-d-lysine-coated (#P7280, Sigma) glass coverslips (#1001/18, Glaswarenfabrik Karl Hecht GmbH & Co) in 12-well plates. The cells were cultured in Neurobasal G3 medium (#21103, Gibco), supplemented with B27 (#17504, Gibco), 10 mg/l gentamicin (#15-750-037, Gibco), 0.5 mM l-glutamine (#G8540, Sigma) and 25 µM l-glutamate (#G1626, Sigma). After 5 days, the G3 medium was exchanged for G2 medium (G3 medium without l-glutamate), and the cells were maintained without changing the media until the 14th day of culture, when the cells were fixed and used for immunocytochemistry.

### Culture of Primary Astrocytes and Microglia

Primary glial cell cultures were prepared from 1-day-old Wistar rat pups, as previously described [13, 14]. Briefly, the forebrains of the pups were aseptically removed and homogenized through mechanical dissociation. The cells were isolated from the cerebral cortices through trypsinization (0.025 % trypsin at 37 °C for 20 min) and mechanically dissociated into a single cell suspension. The cells were plated onto poly-l-lysine-coated (#P4707, Sigma) 75-cm^2^ culture flasks at a density of 3 × 10^5^ cells/cm^2^ in culture medium comprising DMEM with GlutaMAX and high-glucose at 4.5 g/l (#31966-021, Gibco), supplemented with 10 % fetal bovine serum (FBS, #10500-064, Gibco), penicillin (100 U/ml) and streptomycin (100 µg/ml) (#15140-148, Gibco). The cells were cultured at 37 °C, 95 % O_2_ and 5 % CO_2_. The culture medium was changed after 3 days and then every 4–5 days. After 9–10 days in culture, the confluent glial cells were shaken and centrifuged to recover microglia from astrocytes and oligodendrocytes in culture. The loosely adhered microglia were recovered through mild shaking and plated at a density of 2–3 × 10^5^ cells/cm^2^ in 24-well plates or 60-mm dishes. After changing the media to remove the nonadherent cells, the adherent microglia were incubated for 48 h.

To remove oligodendrocytes from astrocytes, primary glial cells in 75-cm^2^ culture flasks were placed on a rotary shaker and shaken at 37 °C for 24 h (200 rpm). The culture medium containing oligodendrocytes was removed, and the astrocytes were plated onto 10-cm^2^ culture dishes. After 3 days, the astrocytes were trypsinized and plated onto poly-l-lysine-coated glass coverslips (#1001/18, Glaswarenfabrik Karl Hecht GmbH & Co) in 12-well plates at a concentration of 75  ×10^3^ cells/ml and maintained in culture for 72 h, followed by fixation and immunocytochemistry.

### Scratch Assay

Astrocytes (10^5^ cells) were seeded onto 18 mmØ glass coverslips pretreated with a solution of 100 µg/ml poly poly-l-lysine (Sigma) and incubated in astrocyte culture medium until reaching confluence. Confluent monolayers were scraped with a 200 µl pipette tip. Cells were fixed in 4 % paraformaldehyde in PBS with 4 % sucrose 6, 24 or 48 h following wounding. Immunocytochemistry for Ttyh1 and GFAP was performed as described below.

### Culture of Oligodendrocytes

Rat oligodendrocyte precursor cells (OPCs) were prepared according to a standard shaking method based on the differential adherent properties of glia [15–17]. Briefly, the forebrains from 1-day-old Wistar rat pups brains were aseptically removed and homogenized through mechanical dissociation. The cells were plated onto poly-l-lysine-coated (#P4707, Sigma) flasks, maintained for 10 days in DMEM/F12 (containing 10 % fetal calf serum, 2 mM glutamine and antibiotics) and fed every 2–3 days by a complete media change. At 10 days after plating, the culture flasks were placed onto a rotary shaker and pre-shaken at 37 °C for 6 h to remove the microglia cells. The medium was changed, and the flasks were shaken at 37 °C for an additional 18–20 h. The cell suspension containing OPCs was collected through centrifugation. The cell pellet was suspended in OPC medium SATO medium containing DMEM with GlutaMAX (#31966-021, Gibco) supplemented with 100 μg/ml BSA, 6.2 ng/ml progesterone, 16 μg/ml putrescine, 5 ng/ml sodium selenite, 400 ng/ml T4, 400 ng/ml T3, 50 μg/ml holo-transferrin, 5 μg/ml insulin, 10 ng/ml PDGF, 10 ng/ml FGF, penicillin (100 U/ml) and streptomycin (100 µg/ml)] and plated onto poly-l-lysine-coated glass coverslips (#1001/18, Glaswarenfabrik Karl Hecht GmbH & Co) in 12-well plates at a concentration of 2 × 10^5^ cells/ml. The cells were fed every other day with 10 ng/ml PDGF and 10 ng/ml FGF until the 8th day of culture. To obtain differentiated oligodendrocytes, the OPCs were cultured for 7 additional days in differentiation medium (DMEM (#31966-021, Gibco) containing 10 % fetal bovine serum (FBS, #10106-151, Gibco), penicillin (100 U/ml) and streptomycin (100 µg/ml) (#15140-148, Gibco)]. The medium was changed every 2–3 days. The differentiated oligodendrocytes were fixed and used for immunocytochemical staining.

### Amygdala Stimulation-Induced *Status Epilepticus*

All animal procedures were approved by the Ethical Committee on Animal Research of the Nencki Institute, and the experiments were conducted in accordance with the guidelines of Directive 2010/63/EU of the European Parliament and the Council of the European Union (EU). Adult male Sprague–Dawley rats (290–320 g) were obtained from the The Mossakowski Medical Research Centre (Polish Academy of Sciences, Warsaw, Poland) and housed in a controlled environment (24 °C, lights on 07:00–19:00) with free access to food and water. *Status epilepticus* (SE) was triggered through electrical stimulation of the amygdala as previously described [18], with some modifications [19]. Briefly, a stimulating and recording bipolar electrode (Plastic One Inc., Roanoke, VA, USA, #E363-3-2WT-SPC) was implanted into the left lateral nucleus of the amygdala under isoflurane anesthesia. For surface EEG recording, a stainless steel screw was implanted contralaterally into the skull over the right frontal cortex. After a 2-week recovery period, the animals were electrically stimulated via the intra-amygdala electrode to evoke SE. Stimulation consisted of a 100-ms train of 1-ms biphasic square-wave pulses (400 µA peak to peak) delivered at 60 Hz, every 0.5 s for 20 min (Master-8 Stimulator connected with an ISO-Flex stimulus isolation unit, A.M.P.I., Israel). SE was terminated at 1.5–2 h after stimulation through the intraperitoneal injection of diazepam (20 mg/kg; Relanium, Polfa SA, Warsaw, Poland). The sham-operated control animals were implanted with electrodes but did not receive electrical stimulation. Control (n = 7) and stimulated (n = 7) rats were sacrificed 4 days later. Animals were video-EEG monitored (Comet EEG, Grass Technologies, West Warwick, RI) to confirm development of SE lasting at least 90 min.

### Immunocytochemistry

Primary hippocampal neurons, astrocytes, microglia and oligodendrocytes in culture were fixed for 10 min in 4 or 2 % paraformaldehyde (#P6148, Sigma) in PBS containing 4 % sucrose (#S0389, Sigma), permeabilized with 0.5 % Triton X-100 (#X100, Sigma) in PBS for 15 min, blocked (60 min at RT) in 5 % bovine serum albumin (#A2153, Sigma) (BSA) in 0.25 % Triton X-100 in PBS, and incubated overnight at 4 °C with primary antibodies diluted in PBS containing 3 % BSA and 0.25 % Triton X-100. Subsequently, the cells were washed in PBS and incubated (2–2.5 h) with secondary antibodies diluted in PBS containing 3 % BSA and 0.25 % Triton X-100. The cell nuclei were counter-stained with 4,6-diamidino-2-phenylindole, DAPI (1:1000, #D9542, Sigma). Each staining was performed using 3–5 independent cultures.

### Immunohistochemistry

The rats were anesthetized with morbital (1.25 ml/kg, i.p.) and perfused with 0.9 % NaCl (2 min, 30 ml/min), followed by perfusion with 4 % paraformaldehyde in 0.1 % phosphate buffer (PB; 0.1 M Na_2_HPO_4_, and 0.1 M NaH_2_PO_4_, pH 7.4) for 20 min (30 ml/min) as previously described [11]. Next, the brains were removed, postfixed for 4 h in the same fixative, cryoprotected in 30 % sucrose in 0.02 M PB buffer for 3–4 days, frozen on dry ice and stored at −70 °C. Coronal sections (30 µm) were cut and stored in a cryoprotectant solution (30 % ethylene glycol, 25 % glycerol, 0.05 M PB) at −20 °C.

Immunohistochemistry was performed on free-floating coronal brain sections using a standard procedure [11]. Briefly, for double immunofluorescence staining, after blocking for unspecific binding for 2 h with PBS containing 3 % bovine serum albumin (BSA, Sigma) and 0.1 % Triton X-100, a mixture of primary antibodies (diluted in PBS containing 0.3 % BSA and 0.01 % Triton X-100) was applied, and the cells were incubated overnight at 4 °C. Subsequently, the sections were incubated for 2 h with the appropriate secondary antibodies diluted in PBS (containing 0.3 % BSA and 0.01 % Triton X-100), and the nuclei were counter-stained with 4,6-diamidino-2-phenylindole (DAPI), mounted on gelatin-coated slides and coverslipped in Vectashield (Vector Laboratories). For detection of Ttyh1 with 3,3′-diaminobenzidine (DAB) as a chromophore, sections were incubated with anti-Ttyh1 antibody, and then in a solution of appropriate biotinylated antibody. For signal detections, sections were incubated in avidin–biotin solution (ABC kit Vectastain, #PK6100, Vector Laboratories) and then in 0.05 % 3,3′-diaminobenzidine (DAB) solution containing 0.04 % H_2_O_2_.

### Antibodies

The following primary antibodies were used in the present study: mouse anti-Ttyh1 (1:200, #WH0057348M4, Sigma), rabbit anti-Synaptotagmin 1 (1:100, #105102, Synaptic Systems), rabbit anti-Synaptoporin (1:500, #102002, Synaptic Systems), rabbit anti-Neuronal Class III β-Tubulin (1:750, #PRB-435P, Covance), rabbit anti-LAMP1 (1:500, #ab24170, Abcam), rabbit anti-GRP78 (1:200, #ab21685, Abcam), rabbit anti-Calnexin (1:40, #ab22595, Abcam), rabbit anti-GM130 (1:200, #ab52649, Abcam), rabbit anti-TGN46 (1:500, #ab16059), rabbit anti-Clathrin (1:500, #ab21679, Abcam), rabbit anti-Rab5 (1:500, #ab18211, Abcam), rabbit anti-Rab11 (1:100, #71-5300, Invitrogen), rabbit anti-APPL2 (home made, [20]), rabbit anti-Olig2 (1:500, #AB9610, Chemicon Millipore), rabbit anti-Iba1 (1:1,000, #019-19741, Wako), mouse anti-GFAP-Cy3 (1:1,000, #C9205, Sigma). Anti-IgG mouse (1:200, #553447, BD Pharmingen) anti-IgG rabbit (1:200, #ab46540, Abcam) were used as controls to test specificity of immunostainings. The following secondary antibodies were used in the present study: goat anti-mouse conjugated with Alexa Fluor 488 (1:200, #A11001, Molecular Probes), goat anti-rabbit conjugated with Texas Red (1:200, #T2767, Molecular Probes), horse anti-mouse conjugated with Texas Red (1:200, #TI2000, Vector Laboratories), and anti-mouse biotinylated antibody (1:200, #BA2001, Vector Laboratories).

### Western Blot

Proteins isolated from rat primary cultured neurons, astrocytes, microglia and rat brain tissue were processed for western blot analysis using a standard protocol [11]. The experiment was repeated three times. Briefly, the cells were lysed in lysis buffer containing 50 mM KCl, 50 mM PIPES, 10 mM EGTA, 2 mM MgCl_2_, 0.5 % Triton X-100, 100 μM PMSF and 1 mM DTT. The brain tissue was homogenized in the same buffer using a TissueRuptor homogenizer (Qiagen). The lysates were centrifuged at 20,800*g* for 20 min at 4 °C. The supernatants were collected and stored at −20 °C. The protein concentration was determined using Protein Assay Dye Reagent (#500-0006, BioRad). The samples were separated using SDS-PAGE and transferred to nitrocellulose (#RPN303D, Hybond™-ECL, Amersham). For immunodetection, the nonspecific binding was blocked through incubation in 5 % non-fat milk in TBST (0.5 M Tris, 0.9 % NaCl, 0.1 % Tween 20, pH 8). Subsequently, the membrane was incubated in a solution of mouse anti-Ttyh1 antibody (1:500, #WH0057348M4, Sigma) in TBST overnight at 4 °C, followed by incubation in a solution of secondary anti-mouse horseradish peroxidase-linked antibody (1:5,000, #NA931, Amersham Biosciences) in TBST for 2 h at RT. Chemiluminescent detection was performed using ECL (#RPN2209, Amersham) according to the manufacturer’s instructions. The protein molecular weights were estimated using prestained protein markers. To verify that equal amounts of protein were loaded, the membrane was stripped and reprobed with mouse anti-GAPDH antibody (1:300, #MAB374, Chemicon Millipore) in TBST overnight at 4 °C, followed by incubation with secondary anti-mouse horseradish peroxidase-linked antibody.

### Image Acquisition and Analysis

The Carl Zeiss LSM780 Spectral Confocal System interfaced with Axio Observer Z.1 inverted microscope equipped with Plan Apochromat 40×**/**1.4 Oil DIC, Plan Apochromat 63×**/**1.4 Oil DIC objectives, Ar ion (488 nm), DPSS diode (561 nm), diode (405 nm CW/pulsed) laser and Zen 2011 software were used for fluorescence image acquisition.

The images were processed using ImageJ software (Ras-band, W.S., ImageJ, U. S. National Institutes of Health, Bethesda, Maryland). The displayed images were not manipulated beyond adjusting the histogram. Deconvolution was performed using Huygens Software (SVI, Hilversum, The Netherlands). Colocalization analysis was performed with ImarisColoc (Bitplane, Zurich, Switzerland). Every time colocalization channel containing only colocalized voxels was generated. Results are presented as a merge of fluorescent channel and colocalization channel for easier perception of colocalization sites. Pearson’s correlation coefficient was calculated to quantify the degree of colocalization between fluorophores. Ttyh1 vesicle volume analysis was performed using Spots algorithm based on absolute intensity included in Imaris software. All the analysis were performed using at least five images, which were obtained from 3 to 5 independent experiments.

Microscope pictures of DAB stained slices were taken using a Nikon Eclipse 80i microscope equipped with a 10× and 20× objective and Wikom Evolution WF camera.

## Results

### Expression of Ttyh1 Protein in Neurons, Astrocytes, Microglia and Oligodendrocytes In Vitro

**Fig. 1:**
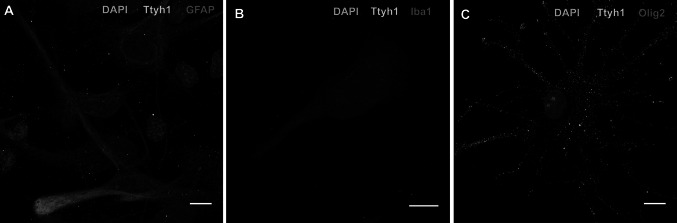
Localization of Ttyh1 protein in rat astrocytes, microglia and oligodendrocytes in vitro. **a** Double immunolabeling of primary astrocytes for Ttyh1 and GFAP. Ttyh1-positive structures were present in the entire cell, in astrocyte somata and in the branches along the filament network stained with GFAP. **b** Ttyh1 immunoreactivity in microglia double-immunostained with anti-Ttyh1 (*green*) and anti-Iba1 (*red*). Fine Ttyh1-positive, dot-like structures were present throughout the entire cell. **c** Ttyh1 immunoreactivity in oligodendrocyte immunostained with anti-Ttyh1 (*green*) and anti-Olig2 (*red*). Ttyh1-positive structures were present throughout the entire cell, in the soma and in branches. The cell nuclei were counter-stained with DAPI (*blue*). *Scale bars* 10 µm. The images represent maximum intensity *Z* projections of confocal sections. DAPI, 4,6-diamidino-2-phenylindole; GFAP, glial fibrillary acidic protein; Iba1, Ionized calcium binding adaptor molecule 1; Olig2, oligodendrocyte lineage transcription factor 2 (Color figure online)

Previous studies have shown the expression of *Ttyh1* mRNA in rat cortical neurons and the widespread expression of Ttyh1 protein in neurons in vitro and in vivo [10, 11]. In the present study we evaluated the expression of Ttyh1 protein in glial cells (astrocytes, oligodendrocytes and microglia) cultured in vitro. Immunocytochemistry with the anti-Ttyh1 antibody revealed the abundant expression of Ttyh1 in astrocytes, oligodendrocytes and microglia in vitro. Pattern of expression was consistent from culture to culture. Images of representative cells are presented on Fig. 1.

In astrocytes and oligodendrocytes Ttyh1 was present in dot-like structures in cell somata and in branches. Similarly, in microglia Ttyh1-positive structures were evenly distributed throughout the cytoplasm. Ttyh1 immunoreactivity in astrocytes and microglia was weaker when compared with oligodendrocytes. We have observed differences in the size of the Ttyh1-immunopositive dots in all cell types.

**Fig. 2:**
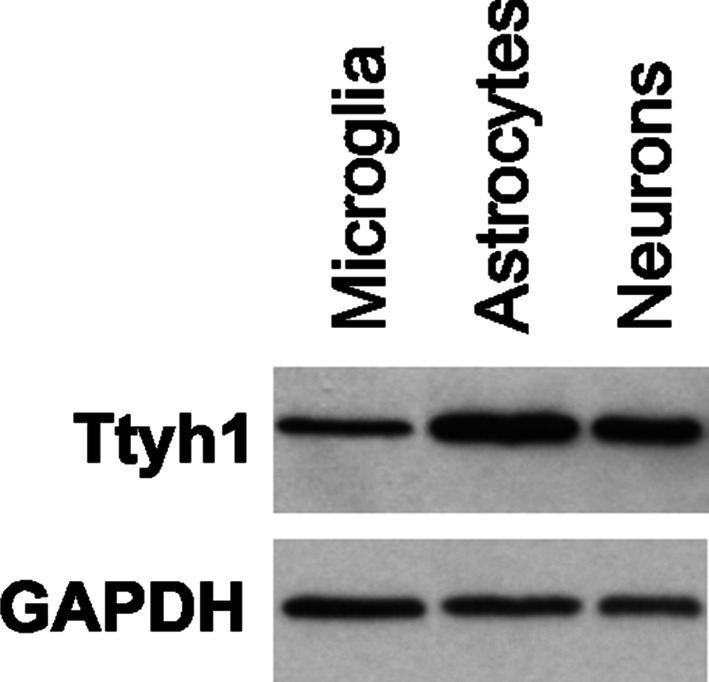
Western blot analysis of Ttyh1 protein expression in protein extracts isolated from microglia, cerebral astrocyte cultures and hippocampal neuronal cultures. A single band represents Ttyh1 protein (predicted molecular weight of 37 kDa), which was detected in all lysates. GAPDH (predicted molecular weight of 38 kDa) was used as a protein loading control. GAPDH—Glyceraldehyde 3-phosphate dehydrogenase

The western blot analysis confirmed the presence of Ttyh1 protein in extracts of isolated hippocampal neurons, cerebral astrocytes and microglia in vitro (Fig. 2).

### Expression of Ttyh1 in the Hippocampus in Control Animals and After Amygdala Stimulation-Induced *Status Epilepticus*

**Fig. 3:**
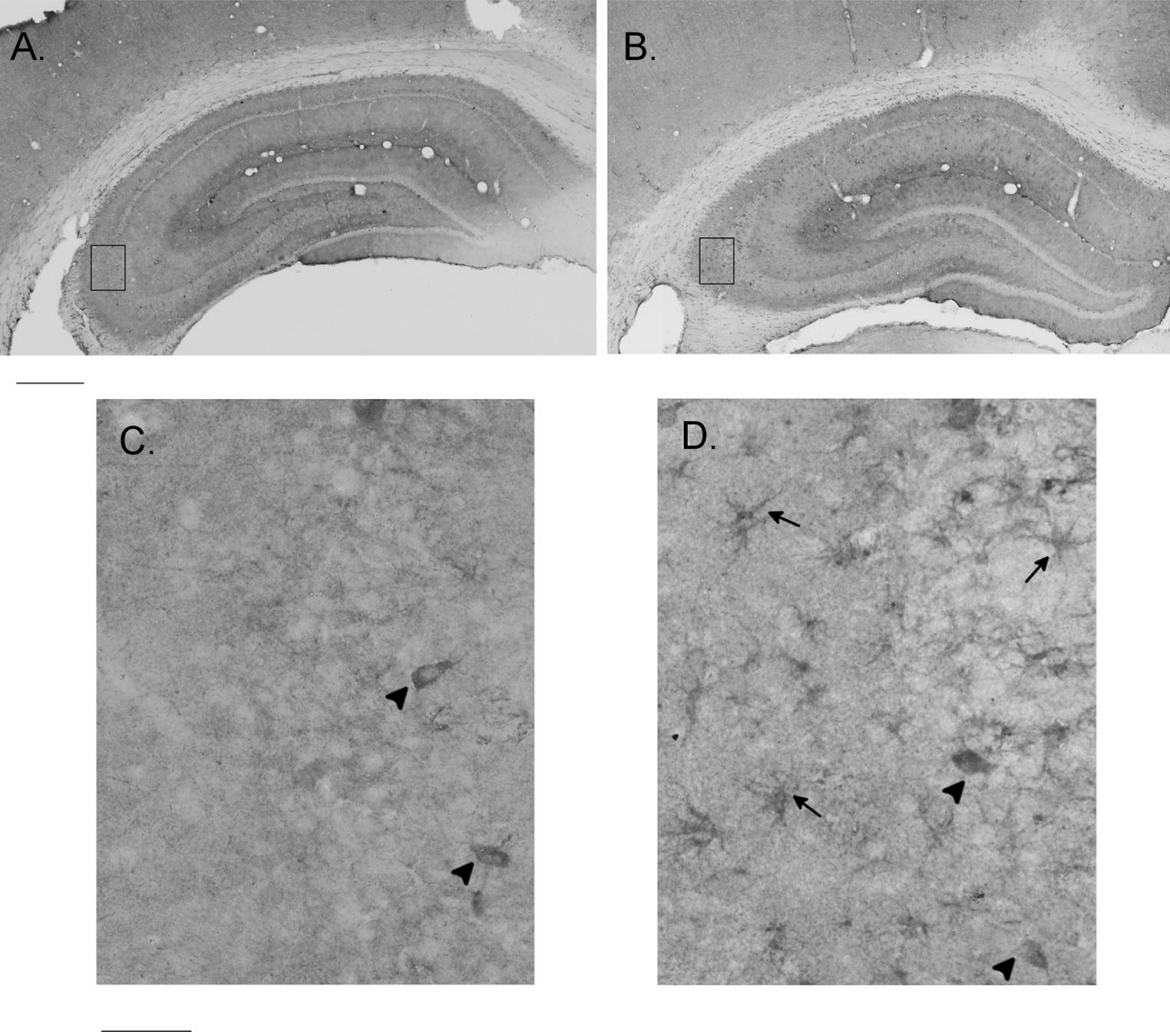
Ttyh1 expression in the normal rat brain and at 4 days after amygdala stimulation-induced *status epilepticus*. DAB immunostaining with anti-Ttyh1 antibody in the hippocampus of a sham control brain (**a**) and at 4 days after *status epilepticus* (**b**). Fragments of pyramidal layer and *stratum oriens* of CA3 region are framed and enlarged in **c** (control brain) and **d** (stimulated brain). In the control brain exclusively neuronal somata within pyramidal layer are stained (indicated with *arrowheads*). At 4 days after *status epilepticus* astrocyte-like profiles (*arrows*) and neuronal somata within pyramidal layer (*arrowheads*) are revealed with immunostaining with anti-Ttyh1 antibody. *Scale bars* 500 µm for **a** and **b**, 50 µm for **c** and **d**

We detected Ttyh1 immunoreactivity in astrocytes, microglia and oligodendrocytes, and this result prompted use to determine whether Ttyh1 is expressed in activated glial cells in vivo. Following amygdala stimulation induced *status epilepticus*, reactive astrocytes in hippocampus can be observed as early as after 24 h and persist for at least 14 days [21]. We performed preliminary DAB immunostainings to detect Ttyh1 protein in slices from rats 24 h, 4 and 14 days after amygdala stimulation-induced *status epilepticus* and time-matched control animals. 4 days following *status epilepticus* majority of immunostained hippocampal cells displayed star-like processes typical of hypertrophic reactive astrocytes. They were more or less evenly distributed among different layers (Fig. 3b, d). In hippocampi of control animals only sparse neuron-shaped somata were strongly labeled (Fig. 3a, c). They were found mainly in the pyramidal layer. In other timepoints we did not observe Ttyh1 expression in cells morphologically resembling astrocytes (data not shown).

**Fig. 4:**
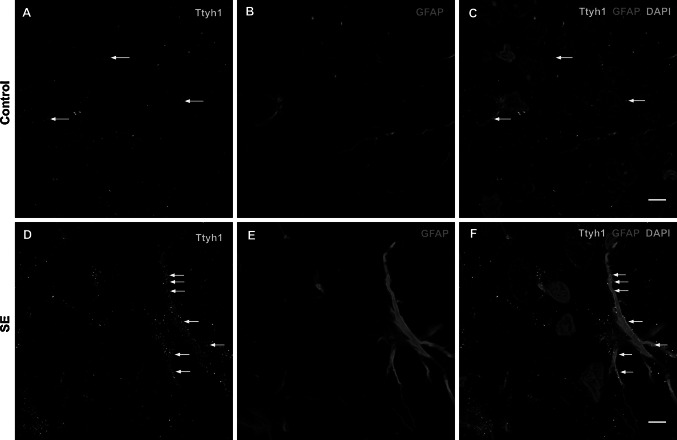
Ttyh1 expression in astrocytes in the normal rat brain and at 4 days after amygdala stimulation-induced *status epilepticus*. **a**–**c** Ttyh1 expression in the hippocampal CA3 of a sham control brain. **a** Confocal image of immunofluorescence with anti-Ttyh1 (*green*) and **b** the astrocyte marker anti-GFAP (*red*), **c** merged image of double immunofluorescence staining demonstrating Ttyh1, GFAP and cell nuclei counter-stained with DAPI (*blue*). **d**–**f** Ttyh1 expression in the hippocampal CA3 pyramidal layer at 4 days after *status epilepticus*. **d** Confocal image of immunofluorescence with anti-Ttyh1 and **e** anti-GFAP, **f** merged image of double immunofluorescence staining demonstrating Ttyh1, GFAP and cell nuclei counter-stained with DAPI. The *yellow arrows* on *green* channel (**a**, **d**) are pointing single, representative Ttyh1-positive structures. The same Ttyh1-positive structure, marked by the *yellow arrows* on merge channel (**c**, **f**), show the colocalization of Ttyh1 and GFAP. Note that in the control brain, colocalization was negligible, while Ttyh1-positive punctuate staining was observed throughout the astrocytes from the brain after *status epilepticus*. *Scale bar* 5 µm. The images represent single confocal sections. DAPI, 4,6-diamidino-2-phenylindole; GFAP, glial fibrillary acidic protein (Color figure online)

To confirm Ttyh 1 astrocytic localization, we performed double immunohistochemistry, using the anti-Ttyh1 and anti-GFAP antibody. Few Ttyh1-positive dots were observed in GFAP-positive astrocytes in the CA3 of the sham control animals. In contrast, numerous Ttyh1-immunopositive elements were detected in GFAP-positive cells in the CA3 of animals subjected to amygdala stimulation (Fig. 4). The increase in Ttyh1 immunoreactivity in reactive astrocytes in the CA3 reflected the increased intensity of immunofluorescence and the increase in the number of Ttyh1-positive dots (Fig. 4d).

To test possibility that *status epilepticus* induces Ttyh1 expression also in oligodendrocytes or microglia, we performed double immunostainings using anti-Ttyh1 antibody and anti-Iba1 (microglia marker) or anti-Olig2 (oligodendrocyte marker) antibody (data not shown). Ttyh1 immunoreactivity in microglia in vitro was negligible in the control brain. Similarly, in the hippocampus of the stimulated brain, Ttyh1 colocalization with Iba1 was only occasionally observed. Ttyh1 immunostaining in oligodendrocytes in vivo was difficult to interpret. Because the oligodendrocytes insulating neuronal axons are thin and the oligodendrocyte marker Olig2 is specifically localized inside the nucleus, not in the branches, it is difficult to distinguish immunoreactivity in oligodendrocytes. We observed only few Ttyh1-positive dots in the close vicinity of oligodendrocyte nuclei in the control brain and after *status epilepticus*.

### Scratch-Induced Changes in Localization of Ttyh1 in Cultured Astrocytes

**Fig. 5:**
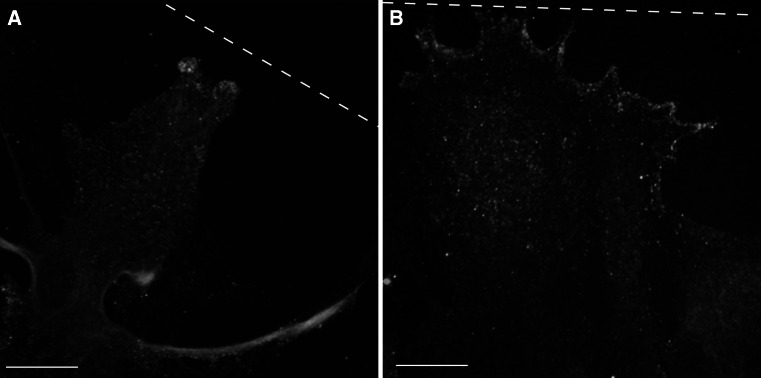
Scratch-induced changes in localization of Ttyh1 in cultured astrocytes. Cultured astrocytes were fixed 48 h after making a scratch and labeled for Ttyh1 (*green*) and GFAP (*red*). *Yellow dashed line* indicates the scratch. In presented astrocytes migrating toward the scratch Ttyh1 was particularly concentrated at the margins of lamellipodia. Ttyh1 formed deposits only in astrocyte processes most protruding toward the scratch (**a**) or along the leading edge (**b**). The cell nuclei were counter-stained with DAPI (*blue*). *Scale bar* 25 µm. The images represent maximum intensity *Z* projections of confocal sections. DAPI, 4,6-diamidino-2-phenylindole; GFAP, glial fibrillary acidic protein (Color figure online)

Reactive astrogliosis, observed in many pathological brain processes including epileptogenesis, can be induced in vitro by a scratch injury [22]. Astrocytes rapidly polarize after lesion and extend their processes perpendicularly to the scratch. We performed Ttyh1 immunofluorescent staining on astrocytes fixed 6, 24 and 48 h after wounding. We observed gradual filling of the area of the scratch by migrating astrocytes. In some migrating astrocytes we observed concentration of Ttyh1 protein at the margin of lamellipodia (Fig. 5). In narrow lamellipodia Ttyh1 was deposited only in the part of the process most protruding toward the scratch. In wider lamellipodia it was concentrated along the whole leading edge. Such uneven distribution of Ttyh1 in astrocytes along the scratch at any timepoint was observed only in fraction of cells.

### Ttyh1 Immunopositive Puncta Volume and Identity in Cultured Neurons and Astrocytes

In neurons and in glial cells Ttyh1 is present in form of dot-like structures of different sizes. Hypothetically this heterogeneity may reflect presence of Ttyh1 in different subcellular structures. Neurons and astrocytes are particularly interesting in terms of Ttyh1 expression, as in those types of cells we were able to detect prominent Ttyh1 immunoreactivity in vivo. We asked whether in those two cell types Ttyh1 is present in the same subcellular structures. Firstly, we addressed the issue indirectly. We compared distribution of Ttyh1 immunopositive puncta volume in cultured neurons and astrocytes. Secondly, we tried to colocalize Ttyh1 with different subcellular markers in neurons and astrocytes.

**Fig. 6:**
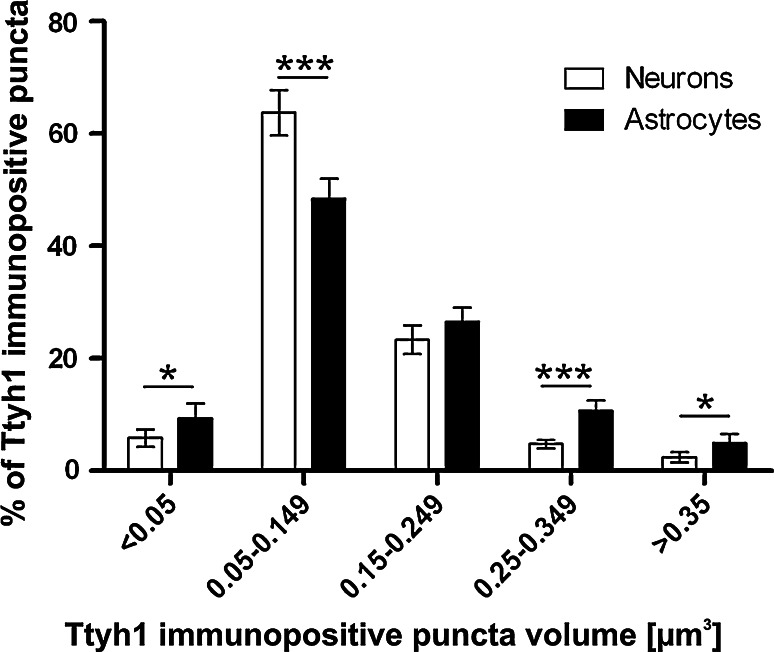
Analysis of distribution of Ttyh1 vesicle volume in cultured neurons and astrocytes. Volume of Ttyh1 immunopositive puncta was determined using Imaris software. Immunopositive puncta are classified into groups depending on their volume. Percent distribution of Ttyh1 vesicle volume in both neurons and astrocytes is similar, with most puncta having volume 0.05–149 µm^3^ and only few percent of puncta bigger than 0.35 µm^3^. Note that proportions of immunopositive puncta in different size groups vary between neurons and astrocytes. Neurons, n = 5; astrocytes, n = 5; *t* test, *p < 0.05; ***p < 0.001

We classified all Ttyh1 immunopositive puncta into one of five groups depending on their volume (Fig. 6). Distribution of Ttyh1 immunopositive puncta volume was roughly the same for neurons and astrocytes, with relatively small proportion of <0.05 µm^3^ puncta, highest proportion of 0.05–0.149 µm^3^ puncta and gradually decreasing contribution of 0.15–0.249, 0.25–0.349 and >0.35 µm^3^ puncta. However, percentages of puncta in different size groups varied between neurons and astrocytes as the puncta 0.05–0.149 µm^3^ were more abundant in neurons than in astrocytes, while small puncta of <0.05 µm^3^ and the large ones (0.25–0.349 and >0.35 µm^3^) were more abundant in astrocytes.

**Fig. 7:**
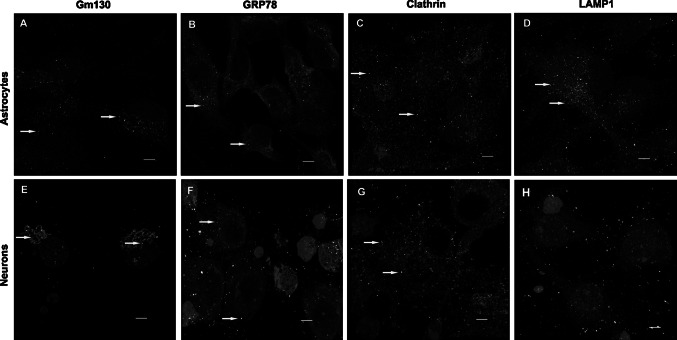
Ttyh1 colocalization with subcellular organelle markers in primary cultures of hippocampal neurons and cortical astrocytes. The panels represent confocal images of immunofluorescent staining with anti-Ttyh1 (*green*) and antibodies directed against different organelle markers (*red*) and cell nuclei counter-stained with DAPI (*blue*). Intensity correlation-based analysis was performed on Imaris software. The colocalized areas, representing the overlay of *green* and *red pixels*, are shown as white dots (see *yellow arrows* that are pointing representative examples of *white dots*). The colocalization of Ttyh1-positive dot-like structures with the Golgi apparatus marker GM130 (**a**), endoplasmic reticulum marker, GRP78 (**b**), coated pits and vesicles marker, clathrin (**c**) in neurons and astrocytes. Ttyh1 colocalizes with LAMP1 (late endosome-lysosome marker) in astrocytes (**d**) and does not colocalize in neurons (**h**). *Scale bar* 5 µm. The images represent single confocal sections. GM130, 130-kDa *cis*-Golgi matrix protein; GRP78, 78-kDa glucose-regulated protein; LAMP1, lysosomal-associated membrane protein 1 (Color figure online)

In a previous study, in which we performed a pull-down assay using hippocampal protein lysates, we revealed that Ttyh1 protein specifically binds to a number of proteins, including a large group of proteins localized in the endoplasmic reticulum or Golgi apparatus [11]. To further characterize the subcellular localization of Ttyh1 protein, we performed double immunocytochemistry on primary hippocampal neurons and primary cortical astrocyte cell cultures using a number of antibodies against specific subcellular markers. Ttyh1 protein has been detected in the Golgi apparatus (Pearson’s correlation coefficient PCC, neurons r = 0.30, astrocytes r = 0.32; Fig. 7a, e), endoplasmic reticulum (PCC, neurons r = 0.27, astrocytes r = 0.47; Fig. 7b, f) and clathrin-coated vesicles (PCC, neurons r = 0.27, astrocytes r = 0.35; Fig. 7c, g) in both neurons and astrocytes. In astrocytes, Ttyh1 colocalized with LAMP1, the marker of late endosomes and lysosomes (PCC, r = 0.48; Fig. 7d). No colocalization of Ttyh1 with LAMP1 was detected in neurons (PCC, r = 0.021; Fig. 7h).

We observed no colocalization of Ttyh1 protein with a number of other cell compartment-specific markers, including markers of early endosomes (Rab5 and APPL2), recycling endosomes (Rab11), trans-Golgi network (TGN46), endoplasmic reticulum membrane (calnexin) and synaptic vesicles (synaptoporin and synaptotagmin 1).

## Discussion

Previous in vivo studies have indicated that in the brain, Ttyh1 protein is abundantly expressed in punctuate, dot-like structures localized in neuropil and neuronal somata [11], and Ttyh1 mRNA in the normal brain is expressed in neurons [10]. Therefore, we concluded that Ttyh1 is predominantly a neuronal protein. However, in the present study, we detected Ttyh1 expression in astrocytes, microglia and oligodendrocytes in vitro and this result prompted use to determine whether Ttyh1 is expressed in activated glial cells in vivo. In amygdala stimulation model of temporal epilepsy neurodegeneration and consequent astrogliosis and microgliosis occur in several brain areas, including hippocampus [21, 23]. In hippocampal slices from rats 4 days after *status epilepticus* we observed Ttyh1 immunoreactivity in cells possessing star-like processes characteristic for hypertrophic astrocytes, while in control brain we observed immunostaining only in neurons. We confirmed astrocytic localization of Ttyh1 protein by collocalizing it with GFAP. We conclude that in physiological conditions Ttyh1 is expressed mainly in neurons, but in brain pathology it may be also be expressed by astrocytes. Previously we described Ttyh1 immunoreactivity in the hippocampi of animals 14 days after *status epilepticus* [11]. At this timepoint we observed increased neuropilar Ttyh1 immunoreactivity in inner molecular layer of dentate gyrus, but we did not detect immunostained astrocytic profiles despite the fact that astrogliosis in epileptic hippocampus is persistent and can be detected at this timepoint [21]. Therefore we hypothesize that astrocytic expression of Ttyh1 is characteristic for early phase of astrogliosis. Despite the lack of Ttyh1 expression in astrocytes in control brains, we have observed Ttyh1 expression in astrocytes in vitro which may indicate that these cells do not maintain a complete resting phenotype in dissociated culture. We did not observe prominent Ttyh1 immunoreactivity in microglia and oligodendrocytes in vivo, either in slices from control rats or from stimulated animals. However we can not exclude possibility that in other pathological conditions microglia and oligodendrocytes express Ttyh1.

We performed a scratch injury on cultured astrocytes monolayer to mimic reactive astrogliosis in vitro. In some cells migrating into the scratched area we observed that Ttyh1 is concentrated at the leading edge of lammelipodia. It has been shown in HEK 293 cell line, that Ttyh1 was similarly concentrated at the peripheral membrane of cells, where it induced formation of filopodia and co-localized with α5 integrin subunit [8]. Integrins are crucial for cell migration and its attachment to extracellular matrix. During the process of attachment of astrocytes, some integrin subunits can be found at the margins of the cell on lamellipodia [24, 25], which resembles pattern of Ttyh1 immunoreactivity in migrating astrocytes. Seizures activate integrin signaling and induce a turnover in adhesive contacts [26, 27], which hypothetically may be linked to increased Ttyh1 expression observed during epileptogenesis and epilepsy [10, 28]. We observed increased Ttyh1 immunoreactivity only in small proportion of migrating astrocytes, so it is possible that Ttyh1 is involved only in specific phase of cell migration and adhesion.

As we have showed in our previous paper [11], primary hippocampal neurons after 8 days in culture show abundant expression of Ttyh1 protein with characteristic differences in size and distribution of Ttyh1-immunopositive dots. We could distinguish two populations of Ttyh1-immunopositive dots: a population of large and a population of small dots. The large Ttyh1-positive dots were localized to sites adjacent to neuronal nuclei, and the small dots were localized along neurites. Here, we used older neuronal cultures (14DIV). In comparison to the younger culture (8DIV), older neurons showed less abundant expression and more homogenous distribution of Ttyh1 dots of different sizes. We conclude that the number and distribution of Ttyh1 immunopositive puncta in neurons in vitro depends on the culture age. Such unequal distribution has not been observed in glial cells in vitro. Percent distribution of Ttyh1 immunopositive puncta volume in neurons and astrocytes was comparable. There were differences between neurons and astrocytes in percentage of immunopositive puncta in specific size groups, but it is difficult to asses whether these differences have any functional meaning.

The analysis of Ttyh1 colocalization with the markers of cellular compartments in neurons and astrocytes in vitro revealed that Ttyh1 is present in several subcellular compartments, including the lumen of the endoplasmic reticulum, the *cis* network of the Golgi apparatus, clathrin-coated pits and vesicles and late endosomes-lysosomes. This result is consistent with that of a previous study characterizing proteins that form complexes with Ttyh1 in the brain tissue [11]. The localization of Ttyh1 in the ER has also been demonstrated in HEK293 and HEK293T cells and in the mouse brain [6, 8]. The presence of Ttyh1 protein in the ER and Golgi may reflect Ttyh1 protein synthesis and post-translational modifications. It has been shown that glycosylation occurs in the Golgi and is important for the proper function of Tweety proteins [5]. A small population of Ttyh1-positive structures has been detected in endocytic vesicles and lysosomes. This interesting observation suggests that Ttyh1 is present in transport vesicles and may be involved in endocytosis and/or exocytosis.

Because Ttyh1 expression is primarily restricted to the brain [8] and Ttyh1 is highly expressed in neurons, we verified whether Ttyh1 is present in synaptic vesicles involved in the storage and subsequent release of neurotransmitters at synapses. We were unable to detect colocalization with the synaptic vesicles markers synaptoporin or synaptotagmin 1 in vitro. This observation is consistent with a previous in vivo study showing the sparse colocalization of Ttyh1 protein with presynaptic markers, and it is reasonable to conclude that the majority of Ttyh1-positive elements are not presynaptically localized in the brain [11]. This conclusion is not in agreement with the results of Morciano et al. [9] who co-isolated Ttyh1 protein with the docked synaptic vesicles. However, it is possible that the immunofluorescence used in the present study was not sensitive enough to detect minute amounts of synaptic vesicle-bound Ttyh1 or that Ttyh1 is present at presynaptic sites only under specific conditions.

In conclusion, the data presented in this work indicate the Ttyh1 might be involved in neuronal and glial cell functions. Particularly, the elevated expression of Ttyh1 in astrocytes following damaging brain insult implies some unknown role for Ttyh1 protein in brain pathology. There is little information on the potential molecular function of Ttyh1, thus additional studies are required to determine the precise role of this protein.
